# Metabolic reprogramming and its clinical application in thyroid cancer

**DOI:** 10.3892/ol.2019.10485

**Published:** 2019-06-18

**Authors:** Shi-Shuai Wen, Ting-Ting Zhang, Di-Xin Xue, Wei-Li Wu, Yu-Long Wang, Yu Wang, Qing-Hai Ji, Yong-Xue Zhu, Ning Qu, Rong-Liang Shi

**Affiliations:** 1 Department of Head and Neck Surgery, Fudan University Shanghai Cancer Center, Shanghai 200032, P.R. China; 2 Department of General Surgery, Τhe Third Affiliated Hospital of Wenzhou Medical University, Ruian, Zhejiang 325200, P.R. China

**Keywords:** thyroid cancer, metabolism, prognosis, predictive factor, targeted therapy

## Abstract

Warburg found that tumor cells exhibit high-level glycolysis, even under aerobic condition, which is known as the ‘Warburg effect’. As systemic changes in the entire metabolic network are gradually revealed, it is recognized that metabolic reprogramming has gone far beyond the imagination of Warburg. Metabolic reprogramming involves an active change in cancer cells to adapt to their biological characteristics. Thyroid cancer is a common endocrine malignant tumor whose metabolic characteristics have been studied in recent years. Some drugs targeting tumor metabolism are under clinical trial. This article reviews the metabolic changes and mechanisms in thyroid cancer, aiming to find metabolic-related molecules that could be potential markers to predict prognosis and metabolic pathways, or could serve as therapeutic targets. Our review indicates that knowledge in metabolic alteration has potential contributions in the diagnosis, treatment and prognostic evaluation of thyroid cancer, but further studies are needed for verification as well.

## 1. Introduction

Thyroid cancer is a common malignant tumor with a sharp increase in incidence worldwide (1). Thyroid cancer mainly includes four types: Papillary thyroid carcinoma (PTC), follicular thyroid carcinoma (FTC), medullary thyroid carcinoma (MTC), and anaplastic thyroid carcinoma (ATC) (2). PTC accounts for the largest component among them (>90%). The prognosis of thyroid cancer is closely related to its histological type. For example, the 10-year overall survival rate of PTC is estimated to be 98%, while the median survival of ATC is only 3–5 months (3). For thyroid cancer, surgical resectionis the most important treatment method. Different adjuvant treatments are effective for certain pathological subgroups, such as radioiodine for differentiated thyroid carcinoma (DTC) and chemotherapy for ATC.

Although most thyroid cancers have a good prognosis, approximately 10% of patients with well-differentiated thyroid cancer have a loss of response to radioactive iodine therapy, and poorly differentiated or undifferentiated tumors are more likely to cause disease recurrence and death (4,5). Therefore, it is necessary to investigate new methods of thyroid cancer treatment. Moreover, we observed that some well-differentiated thyroid cancer cases are significantly more aggressive than others, so it is difficult to predict the patient's course. This heterogeneity of thyroid cancer behavior and the inferior quality of life of patients indicate the importance of identifying prognostic markers.

Over a century ago, Warburg (6) found that tumor cells need more glucose than normal cells and tumor cells prefer glycolysis for glucose metabolism even under oxygen-sufficient conditions, rather than undergo mitochondrial oxidative phosphorylation to produce ATP. This is known as the ‘Warburg effect’. However, the special metabolic mode of tumor cells is not a passive change, but a positive change of expression of heredity to alter the metabolic mode for oncogenesis and neoplasm invasiveness. Tumor metabolism can serve as a potential target for the treatment of thyroid cancer. Moreover, tumor metabolism-related molecules may be a marker for the prognosis of thyroid cancer. Previous findings have shown that the development of thyroid cancer is associated with increased glucose uptake (7). Monocarboxylate transporter (MCT) on the tumor cell membrane and the translocation enzyme TOMM20 on the mitochondrial membrane were reportedly associated with the prognosis of thyroid cancer (8,9). The findings suggest that key molecules in tumor metabolism may be the factors involved in predicting the prognosis of thyroid cancer.

## 2. Cell metabolism in thyroid cancer

### 

#### Multicompartment metabolism of thyroid cancer

Multicompartment mode revealed the translation of metabolic intermediates between different regions of cancer cells. Pavlides *et al* first proposed the ‘Reverse Warburg effect’, whereby cancer-associated fibroblasts (CAF) are induced by cancer cells to shift into aerobic glycolysis and produce L-lactic acid and ketone bodies, which is translated to cancer cells as fuels of oxidative phosphorylation (10). Metabolic coupling between glycolytic fibroblasts and cancer cells promotes tumor growth by increasing cancer cell proliferation and inducing resistance to apoptosis (11).

Transporters that translate intermediates between different compartments are important in the multicompartment mode. A noteworthy transporter is MCT, a class of membrane-bound proteins involved in the influx and outflow of small metabolites, such as lactic acid, and pyruvate and ketone bodies (12). MCT4 is responsible for CAF outputting Lactate. Lactate is then taken up by cancer cells via MCT1 (a two-way transporter) on cancer cells and transported to mitochondria through the mitochondrial outer membrane TOMM20 to produce ATP by oxidative phosphorylation (OXPHOS) (9). Therefore, TOMM20 and MCT1 can be used as biomarkers of OXPHOS and MCT4 can be used as a biomarker of glycolysis.

A high expression of MCT4 in head and neck canceris associated with tumor recurrence and more advanced staging (13). Curry *et al* (14) found that PTC tumor cells exhibit a uniform high expression of TOMM20, but have a low expression in normal thyroid and nodular goiter tissue adjacent to the tumor. There was a statistical difference in the expression of MCT4 in CAF between advanced PTC and non-advanced PTC. In another study on ATC, tumor tissues highly expressed both TOMM20 and MCT1 compared with non-tumor tissues, which was different from PTC (high expression of TOMM20 but low expression of MCT1) (9). The high expression of MCT1 means that it allows more pyruvate and lactic acid to enter tumor cells for high-intensity OXPHOS, leading to significant growth advantages in tumor cells (15). The difference in the expression of MCT1 between ATC and PTC probably explainsthe difference in prognosis.

#### Glucose metabolism

It is well known that unlike normal cells, tumor cells undergo aerobic glycolysis as the main form of glucose metabolism (16). Aerobic glucose metabolism is an inefficient metabolic pathway for the production of ATP. Researchers believe that the proportion of tumor cells in the aerobic glycolysis metabolic pathway is mainly due to its contribution to the proliferation and invasion of cancer cells, and enhancement of cancer cells to fight oxidative damage (16–18).

Nahm *et al* found that the expression levels of glycolytic-related proteins is differentin different thyroid cancer subtypes and is associated with prognosis (19). PTC patients with a high expression of glucose transporter 1 (GLUT1) had a shorter overall survival (OS), and hexokinase II-positive medullary carcinoma patients had a shorter OS and disease-free survival (DFS). MCT4-positive PTC patients had shorter OS than MCT4-negative ones. When GLUT1 and MCT4 were highly expressed, DFS and OS was significantly reduced in patients with poorly differentiated thyroid cancer. Several glycolytic-related molecules haveexhibited an important role in the metabolism of thyroid cancer, such as GLUT1, HK, PKM2 and lactate dehydrogenase (LDH).

#### GLUT1

GLUT1, a unidirectional transporter, is responsible for the transportation of glucose across the plasma membrane of mammalian cells. Extensive research has found that it is expressed in a variety of tumor cells and is associated with prognosis. Haber *et al* analyzed the expression of GLUT1 protein in 38 cases of benign thyroid disease and thyroid cancer (20). The results showed that GLUT1 expression was frequently upregulated in thyroid cancer, but weakly expressed in benign nodules and normal thyroid tissues. Nahm *et al* analyzed 556 cases of thyroid cancer, showing that GLUT1 expression was higher in ATC than PTC and higher in PTC than normal cells (19). They also found that the expression of GLUT1 in FTC was significantly higher than that of follicular adenoma (FA). Kim *et al* found that the expression of GLUT1 gene in ATC was significantly higher than that of differentiated cancer (21). In addition, the expression of GLUT1 in PTC was higher than FTC. The above results indicate that the expression level of GLUT1 may be positively correlated with the invasiveness of thyroid tumors. This is consistent with the results observed in other tumors (22). The phenomenon that ATC has a higher expression of GLUT1 than other types of thyroid cancer is probably due to the fact that ATC has the highest metabolic activity in thyroid cancer (23). Therefore, more GLUT1 is needed to take glucose for metabolism. High proliferative activity of the tumor causes hypoxia. Under hypoxic conditions, the expression of hypoxia-inducible factor-1 (HIF-1) is increased and GLUT1 is the target molecule of HIF-1 (24). Previous studies have demonstrated high HIF-1 nuclear staining in ATC (25), which supports this view. Moreover, ATC is a highly metastatic cancer. Its presence of high expression of GLUT1 is consistent with the known phenomenon that ‘GLUT1 expression of some types of tumors is associated with distant metastasis’ (26,27). In addition, 60% of ATC showed p53 mutations and p53 was involved in glycolytic regulation. GLUT1 is inhibited by wild-type p53, but due to p53 mutation, this regulation is disrupted (28).

#### HK

Hexokinase (HK) is the first rate-limiting enzyme in the glycolytic pathway. There are 4 subtypes of HK in mammals and HK2 has the greatest correlation with malignant tumors (29). Nahm *et al* studied a total of 342 PTC samples and it was found that 50% of the PTC samples containing the *BRAF^V600E^* mutation had higher levels of HK2 (19). According to Hooft *et al*, HK expression in metastatic and primary DTC were similar (30). The expression of HK2 in MTC was lower than that of other thyroid cancer subtypes (19).

#### PKM2

Pyruvatekinase (PK) is one of the main rate-limiting enzymes in glycolysis (31). There are three different subtypes (R, L, M) in human body. PKM is widely distributed in tissues and has two isoforms, M1 and M2. Replacement of PKM2 in tumor cells with PKM1 results in a reversal of the Warburg effect, reduced lactic acid production and increased oxygen consumption (32). Coelho *et al* showed that in two types of human thyroid cancer cell lines, B-CPAP and TPC1, there was higher expression of PKM2 mRNA compared to non-tumor cells (33). There is no difference in PKM1 mRNA levels. However, the total PK activity of B-CPAP was higher than non-tumor cells and TPC1 cell line, indicating that the PK enzymatic reaction is dependent on *BRAF* mutations (33). It is believed that PKM isoform expression and changes of PK activity are associated with increased tumor growth rates. Feng *et al* showed that the expression of PKM2 in human PTC is associated with tumor progression and lymph node metastasis (34). Bikas *et al* also found overexpression of PKM2 in thyroid cancer cells (FTC-133 and B-CPAP) characterized by glycolytic dependence (35).

#### LDH

LDH converts pyruvate produced by glycolysis into lactic acid, which can be transported to the outside of the cell to avoid the accumulation of large amounts of lactic acid inside the cells and form an acidic microenvironment that is beneficial to cancer cells. Another important function of LDH is to oxidize the NADH coenzyme produced by glycolysis to NAD+ to maintain the aerobic glycolysis. Of five isozymes, LDH-A is the one closely related to tumor invasion (36). Coelho *et al* compared two PTC cell lines, B-CPAP and TPC1, and found no difference in LDH-A mRNA expression compared to non-tumor cells (33). However, the two tumor cell lines have a higher LDH activity and lactic acid production rate. Kachel *et al* showed a different result, that LDH-A is overexpressed in FTC and PTC compared to non-tumor tissue, and its level in ATC is even higher (37).

### Amino acid metabolism of thyroid cancer

#### Serine/glycine metabolism

Sun *et al* reported that the expression of serine/glycine metabolism-related proteins in different thyroid cancer types is different through analyzing tissues of different thyroid cancer subtypes (38). The expression is higher in poorly differentiated thyroid carcinoma (PDTC) and PTC, and lower in MTC and FTC. In PTC, it is higher in tissues with *BRAF^V600E^* than those without BRAF mutation. Expression of serine/glycine metabolism-related proteins, including phosphoglycerate dehydrogenase (PHGDH), phosphoserine aminotransferase (PSAT), phosphoserine phosphatase (PSPH), serine hydromethyltransferase (SHMT), and glycine decarboxylase (GLDC), is different in different thyroid cancer subtype as mentioned below. Expression of PHGDH, PSAT1, PSPH and tumor SHMT1 is higher in PDTC and PTC, but lower in MTC. Matrix SHMT1 expression was highest in ATC and lowest in FTC. The expression of PSPH, tumor SHMT1 and matrix SHMT1 was higher in PTC than FTC. *BRAF^V600E^* mutant cells have higher PHGDH, PSAT1, PSPH, tumor SHMT1, and interstitial SHMT1 and GLDC expression than non-mutant cells.

#### Glutaminemetabolism

The three proteins that play an important role in glutamine metabolic pathway are amino acid transporter-2 (ASCT2), a transporter of glutamine into cancer cells; glutaminase 1 (GLS1), which converts glutamine into a glutamic acid; glutamate dehydrogenase (GDH), an enzyme that converts glutamic acid to alpha-ketoglutarate, the latter for the tricarboxylic acid cycle. Kim *et al* performed tissue microarray on 557 patients with different pathological types of thyroid cancer (39). The order of tumor GLS1 and GDH expression from high to low was ATC, PTC, FTC and FA. Tumor ASCT2 expression was higher in MTC but lower in FTC. Tumor GLS1 and tumor GDH expression was higher in the PTC with *BRAF^V600E^* mutation than PTC without the *BRAF^V600E^* mutation. Therefore, the more follicular differentiation, the more prone to low expression of GLS1 and GDH.

In other tumors, glutamine metabolism-related proteins (GLS1, GDH and ASCT2) have also been reported to be associated with tumor invasion (40). The possible mechanism is as follows. First, as mentioned earlier, ATC has higher metabolic activity than other subtypes of thyroid cancer. Glutamine metabolism plays an important role in tumor metastasis (41). Second, ATC has been shown to have higher HER-2 expression (42) and activation of the Wnt β-catenin pathway (43). HER-2 and β-catenin pathways are reported to be associated with increased glutamine metabolism (44,45).

#### BRAF mutation and metabolism of thyroid cancer

Since the first discovery of *BRAF* mutations in human cancer in 2002, its importance has become increasingly apparent in PTC (46). However, its mechanism is still under investigation. It has been observed that *BRAF^V600E^* mutations can lead to changes in tumor metabolism, which may be part of the reason for the worse prognosis of *BRAF* mutations.

In PTC, ^18^F-FDG uptake has been shown to vary with pathological differentiation, although the molecular mechanisms responsible for this are unclear (47). It has also been suggested that the *BRAF^V600E^* mutation is associated with ^18^F-FDG uptake rate and GLUT1 expression rate in PTC (48). Yoon *et al* found that *BRAF* mutations in PTC were significantly associated with ^18^F-FDG PET/CT values (49). In addition, the expression of GLUT1 and GLUT3 in *BRAF*-positive PTCs was significantly increased. Chang *et al* also found that *BRAF* mutation is an independent factor in the uptake of PTC ^18^F-FDG, especially for tumors >1 cm (50). Yoon *et al* showed that, the *BRAF^V600E^* mutation was independently associated with high ^18^F-FDG uptake by preoperative PET/CT in PTC patients, but this relationship was not apparent in PTMC (51). Nagarajah *et al* compared the effects of BRAF mutations on ^18^F-FDG uptake in DTC and PDTC (52). In DTC, *BRAF^V600E^*-positive patients had significantly higher ^18^F-FDG uptake than wild-type *BRAF* patients. In PDTC, only a few tumors were positive for *BRAF^V600E^*, and their ^18^F-FDG uptake was not significantly different from that of wild-type *BRAF* tumors. Nahm *et al* observed that the expression of HK2 and MCT-4 in *BRAF* mutation-positive PTC was higher than that in *BRAF*-negative patients (19). According to research by Sun *et al, BRAF* mutations, not only affect glucose metabolism in thyroid cancer cells, but also affect amino acid metabolism (38). The *BRAF^V600E^* mutant PTC expressed more serine/glycine metabolism-related proteins than wild-type *BRAF*. In summary, PTC with *BRAF^V600E^* mutations have a higher expression of metabolism-related proteins and increased ^18^F-FDG uptake compared to non-mutants. This may explain the reason for PTC with positive *BRAF* mutations having a worse prognosis.

## 3. Tumor metabolism as the target of evaluation and treatment of thyroid cancer

### 

#### Biomarkers for prognosis

In summary, we may draw the conclusion that metabolism-related molecules maybe used as biomarkers for the prognosis of thyroid cancer. None of the tumors with low fibroblast-expressing MCT4 staining showed advanced disease or invasive features. Glycolysis-related proteins such as GLUT1 and LDHA, and glutamine metabolism-related proteins such as GLS1, GDH and ASCT2, are associated with invasiveness and prognosis of thyroid cancer. HK2 is associated with the prognosis of MTC.

#### Glutamine metabolism as a therapeutic target

New targets are needed for the therapy of the radioactive iodine-refractory DTC, ATC and MTC. Current targeted therapies for thyroid cancer are tyrosine kinase inhibitors (TKIs) and monoclonal antibodies (mAbs) that inhibit the tyrosine kinase activity essential for the pathogenesis of thyroid cancer (53). However, clinical trial results of TKIs and mAbs are unsatisfactory, with only partial response rates ranging from 2 to 45% (53,54). A possible strategy is to reduce glutamine metabolic enzyme activity or reduce glutamine uptake. GLS1 inhibitors, including BPTES, CB-968 and CB-839, are in preclinical and clinical trials for the treatment of various tumors. BenSer has been reported to inhibit the proliferation of melanoma cells as an inhibitor of ASCT2 (55). Of note, inhibitors of the glutamine metabolic pathway need further research as a treatment for thyroid cancer.

#### Glucose metabolism as a therapeutic target

Studies targeting tumor glucose metabolism have continued for years, and in the field of thyroid cancer, glycolysis inhibitors such as 2-deoxyglucose have been shown to preferentially target ATC in animal models (56). Radioactive iodine-refractory thyroid tumors may be suitable for metabolic-related targeted therapies because they show positive in ^18^FDG-PET scans (57). Moreover, MCT1 inhibitors are also being tested for the treatment of malignant tumors and may be valuable for the treatment of ATC (58).

OXPHOS inhibitors may be effective anti-cancer drugs in thyroid cancer. Previously, there were no strong OXPHOS inhibitors approved by the FDA. In PTC patients, there are data indicating that the weak OXPHOS inhibitor metform in is active in PTC. Metformin has a higher response rate, as was evidenced in a retrospective cohort of subjects with DTC (59). Metformin induces apoptosis in cancer cells and reduces tumor growth in a PTC xenograft model (60). In DTC patients, a single institutional observation study showed that individuals treated with metformin had smaller tumor size, indicating their potential to inhibit tumor growth. However, the beneficial effects of metformin on thyroid cancer may not be due to mitochondrial effects, but through its insulin sensitization. In addition, metformin may reduce the level of TSH, which in turn inhibits the growth of thyroid cancer cells.

## 4. Conclusion

Although there has been some progress in the study of thyroid tumor metabolism in recent years, there are still many gaps to fill. The current research indicates that oncogenes and tumor suppressor genes directly affect cell energy metabolism, leading to phenotype changes conducive to tumor progression. The differential expression of metabolic-related molecules in thyroid cancer with different prognosis and the association between the degree of expression and prognosis suggest that the prognosis of thyroid cancer maybe predicted viametabolic-related molecules. Treatments and drugs targeting tumor metabolism are also under development, and some of them have achieved phased progress, which is likely to open new pathways for thyroid cancer treatment. Consequently, more investigations will be conducted in this field.

## Figures and Tables

**Table I.** Comparison of expression of metabolism-related molecules between different subtypes of thyroid carcinoma.

Metabolism-related molecules	Comparison between different ssubtypes	Refs.
GLUT1	ATC > PTC > FTC > FA	(19,21)
HK2	PTC with *BRAF^V600E^* > PTC without *BRAF^V600E^*	(19)
	Other subtypes > MTC	
PKM		
PKM2 mRNA	BCPAP and TPC1 > NC	(33)
PK activity:	BCPAP > TPC1	
LDH-A	ATC > PTC and FTC > NC	(37)
Serine/glycine	PDTC and PTC > MTC	(38)
Metabolism-related proteins	PTC > FTC	
	PTC with *BRAF^V600E^* > PTC without *BRAF^V600E^*	
Glutamine metabolism-related proteins		(39)
Tumor and Stroma	ATC > other subtypes	
GLS1 and GDH		
Tumor ASCT2	MTC > FTC	
Tumor GLS1 and GDH	ATC > PTC > FTC > FA	
	PTC with *BRAF^V600E^* > PTC without *BRAF^V600E^*	
TOMM20	ATC > NC	(9,14)
	PTC > NC	
MCT1	ATC > NC	(9)
	PTC < NC	

## Data Availability

Not applicable.

## Abbreviations

PTC: papillary thyroid carcinoma
FTC: follicular thyroid carcinoma
MTC: medullary thyroid carcinoma
ATC: anaplastic thyroid carcinoma
DTC: differentiated thyroid carcinoma
PDTC: poorly differentiated thyroid carcinoma
MCT: monocarboxylate transporter
CAF: cancer-associated fibroblasts
OXPHOS: oxidative phosphorylation
HK: hexokinase
LDH: lactate dehydrogenase
PK: pyruvatekinase
GLUT1: glucose transporter 1
